# Good Health and Moral Responsibility: Key Concepts Underlying the Interpretation of Treatment as Prevention in South Africa and Zambia Before Rolling Out Universal HIV Testing and Treatment

**DOI:** 10.1089/apc.2016.0114

**Published:** 2016-09-01

**Authors:** Virginia Bond, Graeme Hoddinott, Lario Viljoen, Melvin Simuyaba, Maurice Musheke, Janet Seeley

**Affiliations:** ^1^ Department of Global Health and Development, Faculty of Public Health and Policy, London School of Hygiene and Tropical Medicine, London, United Kingdom.; ^2^ Zambia AIDS-related Tuberculosis Project (Zambart), School of Medicine, Ridgeway Campus, University of Zambia, Lusaka, Zambia.; ^3^ Desmond Tutu TB Centre, Department of Paediatrics and Child Health, University of Stellenbosch, Tygerberg Campus, P.O. Box 241, Cape Town, South Africa.; ^4^ MRC/UVRI Uganda Research Unit on AIDS, P.O. Box 49, Entebbe, Uganda.

## Abstract

Gauging community responses to the WHO 2015 recommendation to provide antiretroviral treatment (ART) to all people living with HIV (PLHIV) is critical. There is limited qualitative evidence on the acceptability of this Universal Test and Treat (UTT) strategy or community understanding of the impact of ART on reducing HIV transmission, promoted as Treatment as Prevention (TasP). This article explores early understanding of UTT and TasP in 21 urban communities in South Africa and Zambia in 2013 before a community randomized trial of combination prevention—HPTN 071 (PopART). It draws on participatory research conducted in each community, which carried out group discussions and interviews with 1202 respondents and 203 structured observations. Participants were largely unfamiliar with the concepts of UTT and TasP. They were concerned about an accompanying de-emphasis on sexual behavior change. Treatment and prevention seemed, at first glance, to be experienced separately. With the exception of the prevention of mother-to-child transmission, prevention seldom came into discussions about ART. This was partly because this science had not yet been explained to many and also because it was not an easy fit. Contemplating the link between treatment and prevention, participants emphasized both PLHIV taking care of themselves through good health and preventing disease progression and the moral responsibility of PLHIV to prevent HIV transmission. To avoid igniting moralizing and blaming when introducing UTT and TasP, we should capitalize on the “taking care of yourself” legacy while boosting public responsibility through broad antistigma education and patient empowerment efforts.

## Introduction

Thirty years into the epidemic, HIV is an integrated dimension of daily life in South Africa and Zambia. Over this time, communities have had an astonishing exposure to care, pain, suffering, death, and change.^1^ Since the widespread implementation of treatment in southern Africa from 2006, HIV programming has increasingly moved toward universal uptake of testing and treatment. This transition has been galvanized by growing evidence of the advantages of earlier treatment initiation both for people living with HIV (PLHIV) and to prevent HIV transmission.^2^ The emphasis on reducing HIV transmission increasingly falls under a concept labeled Treatment as Prevention (TasP). This has become a driving ideology for HIV policy,^3–5^ influencing funding, focus, and programs and aligning treatment closer to prevention. The recent WHO guidelines recommend antiretroviral treatment (ART) initiation regardless of immune capability (i.e., CD4 count).

In the context of the anticipated scale-up of ART access, it is important to understand how people living in areas of high HIV prevalence understand the concepts of TasP and Universal Test and Treat (UTT). In Africa, there is a lack of community-based research on the acceptability of TasP, UTT, and other antiretroviral (ARV) prevention technologies (e.g., Pre-Exposure Prophylaxis), particularly about the concerns of implementers and users.^6–9^ As Young and McDaid^8^ observe, “Questions about acceptability [of TasP]…need to be push beyond simple willingness to understand the context within which interventions…might be acceptable and/or preferred” (p. 212).

In this article, we use a social public health approach^10^ to describe the influence of social and historical context on understanding of TasP and UTT in places where access to ART regardless of CD4 count is being introduced, implemented, and used in the HPTN 071 (PopART) trial. The emerging analysis identifies good health and moral authority as two underlying concepts that could shape the uptake of these strategies, with clear implications for acceptability and HIV-related stigma.

Late in 2012, 21 urban areas across Zambia and the Western Cape, South Africa, were selected as communities for a cluster randomized trial, Population Effects of Antiretroviral Therapy to Reduce HIV Transmission [HPTN 071 (PopART)].^11^ This trial measures the impact of a combination prevention package on HIV incidence at the population level. The intervention (2014–17) encourages all residents to test for HIV. Those living with HIV are linked to HIV services located in government health facilities. Condoms, voluntary medical male circumcision (VMMC) for HIV-negative men, and treatment of sexually transmitted infections are promoted (ibid). In 7 of the 21 communities, immediate ART was initially provided for PLHIV, irrespective of CD4 count; in the other 14 communities, PLHIV were offered ART according to national treatment guidelines, which, in 2013, stipulated starting ART when the CD4 count dropped below 350. Due to the revised WHO treatment guidelines (2015), from June 2016 onward, early ART was made available and promoted in all Zambian HPTN 071 (PopART) trial communities. South Africa is likely to follow suit in 2017. This article draws on rapid qualitative research carried out before the HPTN 071 (PopART) intervention implementation.

The aim of this article is to explore how HIV treatment and prevention are understood by local residents across the communities and to consider the implications for the recent expansion of early access to ART.

## Methods

Qualitative research was carried out in each of the 21 communities from December 2012 to May 2013 before the randomization of each community to different intervention packages. Each community is defined as the catchment area of a government health facility where HIV services and ART are available.

Over a period of 12 days in each community, a mix of exploratory qualitative methods was used. These included observations of key places, spaces, and boundaries of relevance to local context and HIV and interviews with groups and individuals (Tables 1 and 2). Drawing on an approach used in three other community randomized trials to rapidly collect data on local context, the methodology aims to allow a comparison between different communities by collecting data systematically across a predefined set of four descriptive dimensions.^12–15^ The participatory techniques applied include drawings and statements on cards, mapping, character cards, HIV concept mapping and pile sorting, free listing, and timelines.^16^ The research is carried out in a sequence that starts with broader observations and narrows down to the more focused discussions, observations, and interviews.

**Table 1.** Group Discussions and Key Informant Interviews

	*Groups (*n*)*		*Participants (*n*)*	
*Activity*	*Zambia*	*South Africa*	*Zambia*	*South Africa*
NHC/comm. reps.	12	4	153	26
HIV specialists	10	8	104	49
Older women	12	6	129	29
Younger women	12	8	136	30
Older men	10	6	80	14
Younger men	12	8	127	29
Gender mixed groups	1	3	5	25
PLHIV	12	5	141	30
Key informant interviews	—	—	65	30
Total group discussions	81	48	940 (567 women)	262 (177 women)

HIV specialists include PLHIV and professional, lay, and alternative health providers.

comm. reps, community representatives; NHC, Neighborhood Health Committee; PLHIV, people living with HIV.

**Table 2.** Structured Observations

	*Activity (*n*)*		
*Observations*	*Zambia*	*South Africa*	*Total (*n*)*
Transport depot	24	18	42
Transect spiral walk	12	9	21
Healthcare facility observation	29	10	39
Market areas	12	8	20
Hair salons/barbers	12	9	21
Drinking places and night observation	24	8	32
Church	12	0	12
Guest house observations	11	0	11
Additional observations	4	1	5
Total observations	140	63	203

Healthcare facility observations include antenatal care clinics, ART clinics, and VMMC.

ART, antiretroviral treatment; VMMC, voluntary medical male circumcision.

The data were collected by graduate social scientists assisted by local resident fieldworkers (in Zambia) and research assistants (in South Africa), with one social scientist and one research assistant/fieldworker carrying out the research in each community. The method was piloted in one community in each country, after which the approach and tools were adjusted.

Key themes explored included places considered relevant to the HIV epidemic, the influence of wealth, poverty, and risk-taking on HIV, reflections on the history of HIV and ART, understanding and ranking of HIV prevention strategies, and listing and ranking of local HIV stakeholders. The HPTN 071 (PopART) trial-specific interventions were also explored, namely views on VMMC, household-based counseling and testing, early initiation of ART, links between treatment and prevention, and the feasibility of the proposed UTT intervention.

All 1202 (745 women, 457 men) participants in either interviews or discussions (Table 1) were adults (aged 18 and over) currently living or working in the communities. Group discussion participants of different genders and ages (older men, younger men, older women, younger women) were selected from different locations in the community. In South Africa, this proved more challenging due to security concerns and ethnic divisions. Subsequently, group discussions in South Africa were often smaller and conducted with more spontaneous groups of people, for example, young men who had gathered near a playing field to watch sport. Prearranged group discussions were also held with residents who had particular expertise and perspective on HIV, recruited through the local health facility, nongovernmental organizations (NGOs), and because they were located and identifiable in the community. These included health committee members, members of NGOs, church leaders, healthcare workers (including community lay volunteers), traditional healers, and PLHIV.

Key informants included health facility staff, private clinicians, church pastors, traditional healers, government representatives, civil society representatives (from nongovernmental/faith-based/community-based organizations), a sex worker, and PLHIV. If possible, discussions and interviews were held away from the health facility to encourage participants to talk more freely about health service quality and more broadly about HIV management. The 203 observations were carried out in places of relevance to HIV and local context (Table 2).

The key topics of HIV treatment and prevention presented in this article were explored both through participatory activities and direct questions. Participants were encouraged to focus on relating responses to their community. Understanding and ranking of HIV prevention strategies were explored initially in group discussions with HIV specialists and then in other community groups. Concept mapping^17^ was used to generate statements, key options, themes, and ranking around a key question—What is HIV prevention in this community?

Outputs were written up through routine debriefing of researchers during fieldwork and an analysis workshop carried out immediately after data collection. All recordings were then transcribed and translated verbatim for finer analysis. ATLAS.ti was used to manage the data based on a coding frame with the broad codes of HIV prevention and Living with HIV (including treatment generally and ART specifically) and HIV-related stigma subcodes of both. This article draws on the finer analysis process.

### Ethical approval

Ethical approval was obtained for this research from the University of Zambia Humanities and Social Sciences Research Ethics Committee, the London School of Hygiene and Tropical Medicine Research Ethics Committee, and the University of Stellenbosch Health Research Ethics Committee. Governmental health authority clearance was also obtained in South Africa and Zambia. Written informed consent was obtained from all research participants engaged in research activities other than observations. Outputs and coded data removed community, place, job title, and person names. A key ethical issue encountered was lack of safety in the field—particularly in South Africa at nights and weekends and around the time of the monthly welfare grants payout.^18^ Precautions taken included researchers working in mixed gender pairs, matching researcher ethnicity to the predominant ethnicity and language of any one community, working closely with governmental and local authorities, referral to appropriate services, and withdrawal from the field or activities in the face of heightened threats (ibid).

### The communities

The population size of the 21 communities is estimated to range from 14,500 to 161,615, with Zambian communities having larger populations. The communities were selected partly on the basis of relatively high HIV prevalence, for which pre-HPTN 071 (PopART) trial estimates ranged between 12% and 23%.^11^ Common features are population expansion and mobility (both daily and transient) and a majority of the population categorized as urban poor. Key infrastructure includes at least one government health facility within walking distance for most residents. Housing is a mix of densely situated formal and informal housing, often with poor waste disposal. High unemployment, migrant or seasonal work, and a bustling informal economy are other common features. More distinctive features include race segregation in South Africa, proximity to international borders in six Zambian communities, and the lack of a welfare state in Zambia.

When asked, “What kind of place is this?” participants initially identified the most salient features as poverty, crime, and substance abuse (both alcohol and, particularly in South Africa, recreational drug use). All three features were linked to transactional and casual sex and teenage pregnancies and were regarded by participants as enhancing HIV risk.

It is in the context of these key physical features and harsh realities—and equipped by social networks, recognition of community action, and existing infrastructure and response—that HIV is navigated by residents. HIV compounds an already long list of everyday challenges for residents and is not always a foremost priority.^19,20^ Analogous to residents of a *favela* in Brazil, they are sometimes impotent in the face of ubiquitous realities.^21^ Typically, participants described these challenges as omnipresent and inescapable. As one Zambian NGO employee described, “[we are] victims in our catchment area.” One older male Zambian participant starkly states: “Hunger greets you each time you enter some of these homes, we wake up and sleep with poverty.” Similarly, a young woman South African participant said, “There's not a person, not a guy that stays in the place that doesn't take *tik* [low-grade methamphetamine often mixed with other substances] … You can't even walk safely in this place anymore because you are scared something will happen to you.”

### TasP: as a concept

In 2013, there was mostly no spontaneous use of or familiarity with the terms UTT, TasP, or Test and Treat, although participants were familiar with other HIV acronyms. For example, a group of Zambian PLHIV asked, “UTT? What kind of animal is that?” Only one Zambian healthcare worker carefully broke it down and described it in her own words, “Universal means something that is broad and all over—testing and treatment are something that are on the large scale.”

Both TasP and UTT have been introduced and advocated under an acronym umbrella, similar to many trends in global HIV policy. Drawing on longitudinal interviews with PLHIV in the UK and South Africa, Squire describes how “clouds of acronyms” were “suffusing interviews,” “an emblem of the acronymic optimism about treating away the pandemic expressed by many medical and policy organizations.”^22^ Qualitative research in Scotland^9^ revealed the same limited awareness of TasP as a branded term as seen in Zambia. Resistance to the term was expressed by national policy makers and study implementers in Swaziland, partly on the basis of clarity and given that they were already moving toward offering this as the best standard of care.^7^

Participants were asked, “Have you heard of ARVs being used to prevent HIV?” In both countries, the first response to this question from participants (including healthcare workers and PLHIV) was usually bemusement, and sometimes even laughter. They often stated they had not heard of it and were hearing of it for the first time. Linking treatment to prevention was initially counterintuitive; if you were living with HIV, then prevention seems less relevant. If you were HIV negative, then treatment seemed less relevant. A South African key informant stated, “no … if I am HIV negative, then I take ARVs, it's not going to assist me.”

Bemusement was quickly followed by discussions, more commonly on the efficacy of ART, testing initiatives, early initiation of ART, prevention of mother-to-child transmission (PMTCT), HIV transmission during sex and birth, the prevention of illness in PLHIV, and the role of PLHIV in preventing transmission. A few participants also spontaneously discussed postexposure prophylactics (PEP), the use of Truvada, and the impact of ARVs on the HIV. Overall, it was mostly PLHIV and HIV specialists (see earlier definition of the latter) who had more detailed information about HIV transmission and the science behind ARVs, with a few exceptions (e.g., older men in one Zambian site). However, most participants were familiar with CD4, viral load, sexual transmission, reducing transmission to children, and adherence to ART. Many discussions also contained curiosity, caution, and confusion about the concept and the importance of PLHIV maintaining health by sticking to particular instructions and moral codes.

In relation to other acronyms linking treatment and prevention, all participants were familiar with PMTCT. Some HIV specialist participants mentioned Prevention with Positives, one of which referred to PWP defined as follows: “PWP—Prevention With the Positive—prevention should start with those who are positive, not to infect others who are HIV negative.”

PEP (a drug regimen taken for a month after an exposure to HIV to reduce the chances of infection and available in health facilities) was familiar to a limited proportion of healthcare workers and PLHIV. It was associated with healthcare workers being exposed to blood, to rape cases, and to unprotected sex. A few Zambian HIV specialist participants identified Truvada as being used for prevention, “to help people not catch HIV,” and for PEP. For others, Truvada was something they heard about on the radio or the internet for use outside Zambia. One healthcare worker in Zambia, for example, said that Truvada was not available at the health facility, but people were discovering that it could be used for prevention through the internet, commenting “hence some promiscuous persons would want to take the medication before sleeping around with different partners.”

Pregnant women living with HIV have been encouraged to take ART since 2002 in both Zambia and South Africa. Zambian participants quickly related the concept of ART as PMTCT. PMTCT was acknowledged to have reduced infant mortality and was strongly advocated with the onus put on a woman to know her status and to come to antenatal care with her partner. Treatment adherence during pregnancy was considered critical. This is captured in a Zambian HIV specialist's comment, “They give birth to a baby, you find the baby is negative because if adherence is 100%, but they are those we see that the mother is HIV positive and the baby is born from the womb with HIV, then no adherence.” In South Africa, while the PMTCT program has been highly effective, there was some skepticism about the long-term impact of PMTCT. Some participants relegated PMTCT to a secondary prevention strategy because, in their view, it had not prevented the mother getting infected.

### Treatment and prevention as distinct categories

Unprompted, treatment and prevention were more often considered as requiring distinct management by participants in both Zambia and South Africa. This is reflected in memories of the history of HIV, discussed using a timeline with participants, and a perceived division of responsibility for treatment and prevention services. Five broad chronological phases were identified in both countries with more detail recalled in Zambia. Treatment (including unavailability of treatment) dominated the first four phases and prevention the fifth, reflecting how the global prevention emphasis has trickled down.

The 1980s were recalled as a period when the first cases of HIV were reported. In the 1990s, treatment was unavailable, health education was limited, and there was little motivation to test for HIV. Vivid memories of family members and friends being frail and bedridden, of death, and of stigma were recalled for this first period. Zambian participants also recollected home-based care organizations (HBC) providing practical and spiritual support. The late 1990s to 2005 were marked by awareness of HIV treatment being available to countries and people with more resources, but unavailable to everyone else. This changed from 2005 onward when ART was available and free. This free provision was regarded as progressive and initial fears about the impact on fertility and the strength of the drugs ebbed. However, residual concerns about sustainability, stigma associated with accessing treatment, and the commitment to lifelong treatment remained. The final phase focused, for the first time, on prevention—particularly VMMC. The possibility of a cure for HIV was also mentioned, with the expectation that akin to ART, a cure would be first only available to those in the West.

It was also evident that in both countries, HIV services were usually associated with either treatment or prevention. The government health facility and key supporting NGOs were associated more with treatment and certain other NGOs associated more with particular HIV prevention initiatives (e.g., couple counseling, mobile HIV testing, door-to-door HIV testing, and VMMC).

### Acceptance of universal testing and treatment

Participants were supportive of everyone testing and knowing their status. Some participants (but not all) linked testing everyone to treatment. As one South African healthcare worker reflected, “We are moving too slowly… if we could test more people …then we can …get more people on treatment.” Only a few participants linked testing everyone to treatment regardless of CD4 count. One Zambian health committee was confused by putting PLHIV on treatment, irrespective of CD4, because they understood that PLHIV with high CD4 were already less likely to transmit HIV because they had less *kadoyo* (meaning insect, as often used in Zambia to refer to the HIV). One Zambian PLHIV recalled hearing on the radio that “ARVs will be given to PLHIV who did not know their status to reduce the chances of those that are positive, but have never been tested, from transmitting the virus to other people.”

When asked more specifically about early treatment for PLHIV, many participants said that in their experience starting ART early rather than late was beneficial. Some then went on to imply that PLHIV who opt not to go on ART are spreading the virus. Holt identifies the same blaming tendency between treated and untreated homosexual men living with HIV in Australia, framing it as a sustained tendency to pit unhealthy against healthy.^23^

### Taking ARVs and boosting the health of PLHIV

In both countries, participants rarely used the term ART. Sometimes they used the term ARVs or RVs, but mostly they referred to ART more obliquely, speaking of the medicine, medication, the pills, and the drugs. In Zambia, multiple euphemisms were often used to talk about ART. These touch on the appearance, being seen taking ART, dependency on ART, and provision of ART. Examples include beans (shape of the pills), joining the choir (lining up for treatment), *kutopin'ga* (a play on topping up mobile phone talk time), and government's drugs.

These euphemisms are considerably more pragmatic and optimistic than other terms still circulating in local discourse for PLHIV, which are overwhelmingly derogatory, touching on death, physical frailty, and improper sexual behavior. Many of these terms predate the availability of ART. For example, the euphemism *kanayaka* has been widely used since 2002.^24^ Meaning the light is on, *kanayaka* indicates the moral, physical, and social exposure of PLHIV. This use of euphemisms in Zambia provides an indirectness, which can both protect and obliquely expose PLHIV since they provide doubts about what, exactly, they mean.^22^ In South Africa, in some descriptions of ART initiation, the box of ARVs was equated with a casket (coffin), symbolizing the end of PLHIV's ability to live without medical sustenance.

By 2013, it is evident that ART—or the medicine—was widely acknowledged in both countries as both lifesaving and life-prolonging, allowing PLHIV to regain health and resume social and livelihood activities. PLHIV in Zambia reflected on this, “The coming of this medication has brought life to us.… ARVs have done a great job! We used to die a lot; we used to die when one is not supposed to die. The coming of this medication has brought life to us.” A South African health worker exclaimed, “They [ARVs] pick up someone who was bedridden and get them to use their feet again… there is nothing better than ARVs.” This Lazarus effect and deep appreciation of ART have been widely documented in sub-Saharan Africa.^25,26^

Aside from ART, across all communities, other forms of medicine and healing were also used for managing HIV by some PLHIV. The alternative approaches are used with the aim of boosting immune systems and spiritual life as well as to help manage side effects. These alternatives, revolving around faith healing and herbal remedies, focused more on health, symptom treatment, and cure than prevention. However, the moral principles of religious faith linked treatment and prevention. Faith was regarded as a form of protection from HIV because church goers were supposed to have certain moral behaviors (e.g., fidelity), which lower HIV risk. An older Zambian woman professes this logic, “Follow God's teachings and all those that are following these teachings are protected from HIV.” Faith healing, linked to Pentecostal church movements^27^ and especially in Zambia, was also sometimes claimed to cure HIV through healing sessions (often all night) with prayers and cleansing rituals using water, anointed oil, milk, salt, and stones. Some pastors were said to recommend throwing away ARVs.

Traditional healers also sometimes claimed to have a cure for HIV, but were less likely to instruct PLHIV to stop taking ARVs and more likely to refer to health services. Herbal remedies, sometimes referred to as immune boosters and a mix of local herbs and substances from outside the countries, were marketed and used in all communities. In South Africa, these included *magogotha* (herb), Eastern Cape foods (traditional herbs), seafood tablets, African potato, herbal detox to cleanse the blood, and marijuana.

Taking ARVs was accompanied by specific instructions pertaining to alcohol use and diet. Healthcare workers in both countries also put emphasis on good nutrition and taking medicine. This pressure to take food with ART resounds with hunger being an experienced side effect of ART.^28^ One South African PLHIV explained, “When you take the pills on an empty stomach, they make you sick and kill us.” The poignancy of this hunger in the context of poverty is more acute in Zambia where there is no disability grant or nutrition schemes such as those found in South Africa.

### Confusing TasP with prevention of disease progression

Other than PMTCT, most community participants were not familiar with ART reducing onward transmission of HIV. Participants were familiar with ART reducing the virus (viral suppression) and some deduced the possibility of ART reducing transmission. Most participants would infer that TasP meant prevention of disease progression among PLHIV.

Viral suppression as a result of taking ART was familiar to participants. ARVs were understood by some participants to reduce the replication of the virus if there was good adherence. This was sometimes described as ARVs suppressing HIV. A few participants extended this to observations on the implications for HIV transmission. This is reflected in the contemplations of an older Zambian man, “I know that when a person starts taking ARVs and his immune system is weak and the viral load had really dropped, when that person starts taking ARVs, his viral load is boosted and the strength of the virus is reduced and the chance of that person passing the virus is reduced… so I would say to an extent the ARVs also help to prevent the spread of HIV.”

The notion of undetectable viral load could be perplexing. Older South African women pondered; “They say sometimes when you take your pills right the virus becomes undetectable in the body, what causes that? People say there is no virus anymore and they want to stop taking the pills.” Indeed, ARVs were not believed to cure HIV. For a few participants, the fact that ARVs did not cure HIV was a link with prevention. For example, one South African healthcare worker retorted, “Because it [ART] doesn't cure the HIV, it is prevention.”

HIV specialists were usually the participants most familiar with biomedical explanations of ART reducing HIV transmission. HIV specialist Zambian participants from two communities involved in related HIV research expounded, “It is scientifically proven like nowadays that you can't, you can't pass the virus to another person,” later qualifying, “If someone is on ART, they cannot pass the virus.”

Only in one Zambian community did any participants recall the specifics of this science where a group of PLHIV recalls, “Research being carried out about two years ago … it was found that tenofovir reduces HIV in the body by 90% and the research was done on homosexuals and it was found that the drugs was 90% effective in reducing the transmission of HIV.” A South African healthcare worker draws on an analogy of having sex with someone who has a raw untreated sexually transmitted infection (STI); “You see if you get treated with that STI, yes, but I sleep with you that guy will not have an STI, so yes I think the infection will be minimal.” More unusually, one older Zambian man (not an HIV specialist) noted, “ARV is one method of prevention because when the viral load in the blood stream is low, chances of infecting others become low unlike when it is high.” A Zambian HIV specialist mentioned that D has been added to the ABC (abstinence, be faithful, and condom) campaign—“ABCD… [because] ….drugs are also used as HIV prevention these days.”

Alongside suppressing viral replication, ARVs were recognized as boosting the immune system and thereby the health of PLHIV. This boost was deemed necessary when PLHIV had a low CD4 count and/or were seriously ill. Reinfection is commonly understood as PLHIV preventing themselves from being infected with a different strain of HIV by abstaining from sex, consistently using condoms, or by having sex with the same long-term partner. Studies in Kenya^6^ and Swaziland^7^ also documented the latter concerns about reinfection among health workers and PLHIV. Zambian HIV specialists in one community said they discouraged PLHIV having multiple sexual partners partly because of the risk of reinfection. They explained to PLHIV, “You are going to reinfect each other, you are going to reinfect, every day you are receiving or giving out….”

### The legacy of behavior change and the risk of sexual disinhibition

Underlying concerns about using ART as prevention were any contrary implications for sexual control and behavior. This is captured by an older Zambian man who argued, “ARVs cannot prevent HIV if one is taking them, but continues engaging in multiple sexual relationships.” The latter emphasis echoes the legacy of the vigorous promotion of sexual behavior change from the late 1980s to mid-2000 in sub-Saharan Africa.^20,29,30^ The well-known ABC campaign is one example of this. Many participants noted how the focus on sexual behavior change had now ebbed; this was often voiced as “a move away from no sex.”

The more the TasP concept was explored with participants, the more the discussions shifted to a perceived risk of ART driving transmission through lifting risky sex sanctions developed earlier on under this behavior change flag.^29^ A South African key informant talks about girls who are on ARVs and who are big, saying, “Their attitude says that they are HIV negative…they don't have the attitude of sick people who are sick…because they changed from very thin people…maybe people will be more careless you know what I mean,” then going on to express his concern, “people will no longer fear getting HIV because if you get the pills in time you are going to get fit and … gym.” Zambian middle-aged men make a similar claim, “ART contributes to the spread of HIV because most people on ART are having many sexual partners.”

This concern about newer prevention technologies leading to increased sexual risk-taking has been coined as sexual disinhibition.^31^ For example, community concerns about VMMC encouraging circumcised men to have unprotected and casual sex have been documented in a number of sub-Saharan African countries, including Swaziland.^32^ In all communities in both Zambia and South Africa, participant fears about sexual disinhibition also cropped up in relation to VMMC.

### HIV prevention: “taking care of yourself”

Given that ART was generally not associated with prevention, it is important to understand what was understood by HIV prevention. The phrase “Taking care of yourself” was frequently lined up with prevention in both countries. Prevention was often recognized in both Zambia and South Africa as a combination of approaches, echoing the notion of combination prevention now widely advocated.^4^ One South African key informant elaborated, “It's a combination of all of them, we can't really single one out.”

When talking directly about HIV prevention, in Zambia, participants understood the concept of HIV prevention, but more commonly spoke of reducing HIV transmission. Women were said to know more about HIV prevention in Zambia, but men were said to have the upper hand (i.e., the control). A South African PLHIV presented a considered approach to the responsibility of PLHIV in his definition of HIV prevention, “If you are positive to take the necessary precautions to prevent yourself from spreading the disease and also taking care of yourself, taking the treatment and using condoms.” Another South African key informant also conveyed a combination of preventive strategies, “HIV prevention, I think HIV prevention is…you prevent HIV by taking care of yourself. You must condomise, ja, and…if you must, stick to your wife. Stuff like that.”

Some participants pointed out how prevention was necessary because there was, as yet, no cure. Zambian participants listed and explored a greater number of prevention options and were notably more optimistic about preventing the transmission of HIV. Some prevention options had a moral (often Christian) zeal, for example, listing and instilling Christian values. A range of Zambian discussion groups recorded going to church as an explicit prevention option. This is illustrated by young women who explained, “Going to church is very important and at church, there are rules as well that enable a person not to commit sexual sin.”

South African participants recalled fewer prevention options than Zambians. They recognized their biomedical effectiveness, but were more cynical about their uptake, correct and consistent usage, and long-term viability. Fatalism around getting infected was also more dominant in South Africa; as one South African man expressed, “If I was meant to be infected with HIV, then I would be. It's like driving and you get involved in a car accident by hitting an animal that is on the road. There is no avoiding an accident that was meant for you.”

The consensus was that abstinence in particular was an unattainable ideal or—as participants put it—not practiced, despite being promoted and recommended at the community level. HIV specialists in one Zambian community wryly remarked, “The bible says thou shall abstain, but people do not abstain from sex.” As one South African key informant expounds, “Abstinence, of course doesn't work. It doesn't work for them [community members]. It works for preventing, but it will never work for them.” “Be faithful,” however, was considered more realistic; “For me, HIV prevention is just to be faithful with your partner” (South African key informant).

Male condoms were considered one of the primary practical means of prevention, particularly for young men and women. However, within steady sexual relationships, they are regarded as a symbol of mistrust; this has been the case since they were promoted earlier on in the HIV epidemic.^30,33^ Condoms were also promoted among PLHIV by healthcare workers and PLHIV, particularly in Zambia, to prevent reinfection.

Education and testing for HIV emerged as key prevention strategies with an emphasis on staying HIV negative. Sometimes the latter message could overemphasize the difference between being HIV positive and HIV negative. For example, health specialists in one Zambian community listed “stay HIV−, stay HIV+” as a prevention strategy. VMMC strategies were also experienced as underscoring this serodivide^23^ by reaching out to HIV-negative men and often excluding men living with HIV.

Participants in most Zambian communities had detailed knowledge of VMMC and were often curious to learn more about it. This was in stark contrast to South Africa where VMMC was always left out of listing during concept mapping, was hard to even talk about (and discussions about it were notably held in a low voice), and was not known as a prevention strategy. VMMC was largely rejected by members of the black Xhosa-speaking community as medical circumcision infringed on the traditional practices of male circumcision and the cultural significance of entering manhood through this experience. Participants talked about going to the mountains and a strong preference for their own initiation described by one South African healthcare worker as going to the bush.

In at least three Zambian communities, the partial nature of the protection afforded by VMMC was emphasized by participants, and on the grounds that VMMC only lessens the chances of infection and does not offer complete protection, some South African participants claimed that VMMC was disqualified as a prevention strategy.

### Overlooking social response and movements

Structural constraints on ability to navigate prevention and treatment options require particular attention in sub-Saharan Africa. Indeed, while participants in both countries acknowledged the central role and transformative agency of ART, they discussed HIV first alongside other key challenges of the places where they lived. These prevailing challenges—most notably poverty, housing, food security, unemployment, substance abuse, crime, and violence—were sometimes more overbearing than HIV.^19^ They were also explicitly linked to the risk of acquiring HIV by participants through patterns of transactional sex and risky sexual behavior, blaming, marginalization, male hegemony, power imbalance, and fatalism. Hence, they pointed out, “To address HIV, you need to address these other vulnerabilities,” echoing Kalichman, who warns, “The barriers to prevention that we encountered for years are still with us, including stigma, addiction, poverty, apathy, denial, and avoidance.”^4^

Earlier on in the HIV epidemic, before HIV treatment was developed and widely available, there was more effort globally and within both these countries aimed at transforming structures and engendering behavior change with a combination of both community action and state involvement.^20,29,34,35^ Despite the genuine gains of these efforts—for example, patient empowerment, the HBC movement flagged by Zambian participants—there were limitations in the scale and the reach of the approaches,^35^ leaving a vacuum for prevention interventions.^4^ Part of the attraction and relief of new prevention biotechnologies is that the complexities of social realities—so inescapable for participants in these 21 places—can be boxed as nonamenable to HIV advances, with answers for eventual elimination of HIV lying rather in pills, gels, rubber protection, and surgical procedures measured by models and statistics.

Yet, biomedical interventions are unlikely to live up to their promise if social determinants of access to prevention and treatment are not addressed.^3^ As one South African key informant expressed it, “We should avoid one-dimensional ways of trying to put easy solutions on something which is just much more complicated… and if we just look on the surface, we are not going to address the underlying things about people's relationships with one another.” Therefore, one clear risk of the primary focus of prevention narrowing to TasP is that social movements regress, including patient rights and provider ethics.^36^ The danger is that equitable and universal commitment in practice^21^ and in discourse is narrowed to the mere provision of prevention technologies and to a focus on core transmitters (language used by mathematical modelers to refer to at-risk groups or key populations).

### Good health versus moral responsibility: the risk of a slippage from public to individual responsibility?

Participants in Zambia and South Africa were, in 2013, largely unfamiliar with the concept of TasP. They were, however, living with ART as an option for managing HIV and had got wind of a renewed emphasis on prevention with the introduction of VMMC. They were concerned about an accompanying de-emphasis on sexual behavior change. There was also a distant awareness of advances in curing HIV. Treatment and prevention seemed, at first glance, to be experienced separately—with an obvious exception of PMTCT. Prevention seldom came into discussions about ARVs and other forms of treatment and likewise ART did not habitually come into the prevention frame. This was partly because this science had not yet been explained to many of them and they did not, yet, know about it. It was also because it was not an easy fit, with the exception of PMTCT where the individual treatment of pregnant women living with HIV secured the collective well-being of society through HIV-free children. The information about HIV treatment reducing HIV transmission could build on this familiarity with and support for PMTCT and on the wider understanding of HIV transmission and the effectiveness of ART.

This analysis reveals two behavioral threads that connect treatment and prevention—“taking care of yourself” and moral responsibility. Without TasP being familiar, participants reiterated that HIV prevention was about “taking care of yourself” and, by extension, PLHIV taking care of themselves to maintain their health. This meant not only taking ART but also boosting their immune system and protecting themselves and others by using condoms. This taking care is engendered by years of caring for PLHIV, both before and since ART.

However, alongside care, stigma can also sit^37^ with the long timescale of the HIV epidemic, allowing participants to revert to blaming the victims,^3^ despite scientific knowledge.^20^ Scattered throughout their discussions on HIV treatment and HIV prevention were moral overtones about appropriate and proper behavior and the risk of transgression. In South Africa, perhaps partly due to a stronger legacy of individual rights and the presence of a quasi-welfare state, responsibility lay more heavily with the system, whereas in Zambia, responsibility lay more heavily in moral conduct of individuals. In both Zambia and South Africa, but especially Zambia, this prescription for the adoption of moral responsible life^38^ not only had strong Christian backing but had also been at the fore of earlier HIV interventions before ART being available. When introducing the concept of TasP, there was a tendency for participants to blame PLHIV for spreading the virus, making them responsible, in turn, for containing the virus. Field notes from one Zambian community record commando as a nickname for PLHIV who are on ART, explaining community perceptions, “Most of them [PLHIV] know they are HIV positive, but want to infect any other person they come across.” Hence, the term commando because it implies that when they infect others, they are killing them just as commandos who shoot and kill people. People felt, “ART is contributing to the rise of HIV prevalence in the community because it is helping PLHIV to disguise their sickness, hence infecting others” (Fieldnotes, February 14, 2013).

Evidence for TasP programs framing PLHIV as infected vessels and a means of prevention as opposed to people whose individual health matters^39^ has been documented among adolescents living with HIV in Baltimore (ibid) and homosexuals in Australia.^23^ This “responsibilizing effect”^40^ raises profound questions about the role of PLHIV in prevention of the transmission of HIV infection (ibid, 71). Philbin et al. argue that viral loads have come to represent the link between individual health and the public's health.^39^ As a result of this link, individuals have become subject to new models of HIV prevention and are made responsible in an unprecedented way.^39^ Drawing on a qualitative study in Scotland, Young et al.^9^ argue that TasP could increase the burden of prevention experienced by PLHIV (p. 273). Kippax warns that a reframing of responsibilization, which could occur alongside TasP, is likely to be counterproductive (Kippax personal communication). In a recent article, Vernooij et al. examine a spread of responsibility underlying TasP uptake in Swaziland across national and individual levels.^7^ They further demonstrate that the acceptability of TasP can be improved by understanding locally framed responsibilities (p. 12), drawing on the reframing of TasP away from public benefits of reducing transmission to the benefits of early treatment for all and individuals taking responsibility for their own health.^7^

We should, however, avoid historical amnesia.^20^ Scientific and global HIV transitions across the 30 years of the epidemic play a part in reshaping responsibility for HIV and HIV-related stigma. This is not lost on participants in this research. One South African key informant describes, “If it's [HIV] treated as a chronic disease. I mean, we should have done that in the 80 s, then we wouldn't have been here now. Everything is hush, hush. If they just said it's a viral infection, there's no cure at the moment, but then they made the disease so ugly. So people…can't move away from that. Those powers that be, they stigmatized the disease. Now we must sit with the brunt of that.” Listening more carefully to participants suggests that we should be wary of TasP providing new opportunities for stigma and discrimination^40^ and falling back into the us and them scenario that antistigma and human rights initiatives have tried so hard to break down.

Neither on the global stage nor in all 21 study communities was there much current evidence of substantial stigma reduction efforts. Yet, as Nguyen et al. comment, “it is time to move forward, not backward,^3^ and to actively reduce stigma and discrimination for TasP to be safely implemented.”^41^ The 2016 IAS Conference reiterated this point, reviving a stigma and discrimination focus as critical to future endeavors. Given the danger of responsibility sitting too heavily on the shoulders of PLHIV, TasP programs could adopt an approach that advocates coupling the good news that early treatment protects health alongside broader motivations that build on the understanding of PMTCT and shared social action to prevent HIV.
